# Neighborhood Built Environment Measures and Association with Physical Activity and Sedentary Time in 9–14-Year-Old Children in Saskatoon, Canada

**DOI:** 10.3390/ijerph17113837

**Published:** 2020-05-28

**Authors:** Shatabdi Goon, Saija Kontulainen, Nazeem Muhajarine

**Affiliations:** 1 Community Health and Epidemiology, University of Saskatchewan, Saskatoon, SK S7N 2Z4, Canada; shg345@mail.usask.ca; 2 College of Kinesiology, University of Saskatchewan, Saskatoon, SK S7N 5B2, Canada; saija.kontulainen@usask.ca; 3 Saskatchewan Population Health and Evaluation Research Unit, University of Saskatchewan, Saskatoon, SK S7N 2Z4, Canada

**Keywords:** children, physical activity, sedentary time, neighborhood, built environment, audit tools, NALP, IMI, perception, researcher-rated

## Abstract

This study assessed whether perceptual and researcher-rated measures of neighborhood-built environments (BEs) predict device-based multiple activity-related outcomes, specifically: moderate-to-vigorous physical activity (MVPA), light physical activity (LPA), and sedentary time (ST), in children. Eight hundred and sixteen children aged 9–14 years from Saskatoon, Canada, were surveyed on their perceptions of BE, and their PA outcomes were objectively monitored for one week at three different time frames over a one-year period, September 2014 to August 2015. The researcher-rated BE measures were collected by trained researchers using multiple BE audit tools: neighborhood active living potential (NALP) and Irvine Minnesota inventory (IMI), 2009–2010. A multilevel modeling approach was taken to understand BE influences of children’s PA outcomes. Children’s perceived availability of parks and sidewalks predicted a higher accumulation of MVPA and a lower accumulation of ST. Children’s report of the absence of neighborhood social disorder (e.g., threats from scary dogs/people) predicted a higher LPA, while reported concern about crime predicted a lower MVPA. Researcher-rated neighborhood activity friendliness predicted a lower ST, however, researcher-rated safety from crime predicted a higher ST. Perceived BE characteristic were stronger predictors of children’s PA outcomes compared to researcher-rated BE factors.

## 1. Introduction

Regular physical activity (PA) helps children and adolescents improve cardiorespiratory fitness, strength, flexibility, and bone density and reduces the health risk of childhood obesity and other chronic diseases [1]. Yet, many children in Canada do not get the recommended amount of PA and spend the majority of their time engaged in sedentary behavior [2]. Canadian children spend on average 9 h of their weekday leisure time in a sedentary state, [3] well above the Canadian Society for Exercise Physiology’s recommended limit of children’s sedentary time (ST) of no more than 2 h per day [4].

Current trends of PA behavior among children has resulted in a call to study what extrinsic factors shape children’s PA and ST patterns throughout the day. Children’s movement, or lack of it, represents a complex behavior that is influenced by multiple factors including demographic, biological, social, and psychological factors as well as the environments in which they live. Multilevel ecological approaches are widely invoked to guide research, to identify determinants of PA, and to inform interventions. Neighborhood environments with a focus on the role of the built environment (BE) in facilitating or limiting PA levels and ST in children is an important but evolving focus [5]. Researchers have found that both objective BE measures in the neighborhood (meaning those physical characteristics that are measured directly, using a tool, often by assessors/researchers) and perceived measures (what people report about their neighborhoods) are associated with physical activity [6,7]. These findings are by no means conclusive [8,9]. Some studies report that neighborhoods that are more safe have better accessibility to facilities and sidewalks, and encouraged activity were significantly associated with increased PA or decreased ST in children [10,11,12,13]. Other studies report no significant associations between characteristics of the neighborhood built environment and PA behaviors [14,15]. A review by Ding et al. concluded that in only 34 percent of the studies investigating this association in children, a significant positive association was found. In the other 66 percent of the studies, no association could be established [16]. Authors suggest that inconsistent findings across studies may, in part, be explained by the differences in the methods of measurement of the outcome or exposure (e.g., objective vs. perceived).

Addressing the knowledge gaps in this field, the objective of this paper was to investigate how perceived and researcher-rated BE measures are associated with PA and ST in a cohort of 9–14-year-old Canadian children. We hypothesized that, first, children from neighborhoods that are deemed to “promote” activity (e.g., safe, activity-friendly, with good services and facilities) will engage in more PA and less time in a sedentary state; second, that associations between perceived BE attributes and PA and ST would be stronger than from associations between researcher-rated BE attributes and PA and ST.

## 2. Methods

### 2.1. Study Design

This study utilized data from the Smart Cities, Healthy Kids (SCHK, 2009–2012) [3], and subsequent Seasonality and Active Saskatoon Kids (SASK, 2014–2017) studies [17]. The SASK study is a longitudinal cohort study developed in Saskatoon, Canada, to examine the effects of neighborhood BE on child health through physical activity opportunities across all seasons, among children aged 9–14 years. The data on children’s perceptions of BE and device-based (accelerometer) PA outcomes were derived from the SASK Study (September 2014 to June 2015). Participants wore portable accelerometers during all waking hours for up to seven consecutive days at three different times over a 1-year period and children completed detailed surveys about demographics and their BE perceptions. The SCHK data provided researcher-rated measures of neighborhood BE data at a single time point (2009–2010); specific BE characteristics were assessed independently by researchers using two replicable, validated tools (Neighborhood Active Living Potential, NALP and Irvine Minnesota Inventory, IMI) [18,19]. Both SCHK and SASK were approved by the University of Saskatchewan’s Research Ethics Board and the Catholic and public-school boards of Saskatoon. 

### 2.2. Neighborhood Selection, Recruitment, and Study Participants

Participants were grade 5 to 8 children aged 10–14 and their parents who were recruited to the SASK study (*n* = 758) in Saskatoon, Canada; a detailed methodology employed was published previously [18]. In summary, a multi-stage clustered sampling method was used to recruit children from a sampling frame that consisted of all 65 residential neighborhoods in Saskatoon. Of the 82 invited schools, 33 (40.2%) participated in the study. From the participating schools, children and their parents were invited to participate in the study through a written informed consent letter disseminated by home classroom teachers. Of the 4615 eligible students in those 33 schools, 922 (20.0%) students and their parents consented. At the first (September–December 2014), second (January–April 2015), and third (April–June 2015) data collection time points, 58 (7.1%), 59 (7.2%), and 76 (9.3% of the original consenting population) students were lost to follow up (either absent, had moved to a different school or province, or declined to participate further), respectively. The total number of consenting participants at the first, second, and third collection point therefore included 758, 699, and 623 child-parent dyads from 31 schools. The validated SASK questionnaire was administered in each collection period to capture children’s behavior and perception of a range of factors that influence their activity, including household (including family socioeconomic factors), parental, peer, and neighborhood influences on PA behaviors. A total of 656 child-parent dyads that responded to the SASK questionnaire at least once (86.5% of 758 participants present at the first time point) were included in this study.

## 3. Measures

### 3.1. Physical Activity Outcomes

ActiGraph GT3X accelerometer devices (ActiGraph Corp., Pensacola, FL, USA) were deployed through schools to measure light (LPA), moderate (MPA), and vigorous physical activity (VPA) and time spent in sedentary state (Sedentary Time, ST) [17]. Children were visited at their respective schools and were asked to wear the devices on their right hip using an elastic belt every day for seven consecutive days (including sleeping hours), unless entering water. The devices began measuring data at 00:00 on the day following device deployment (i.e., almost a full day after the device was deployed) to minimize the potential for subject reactivity within the first day of wearing the accelerometer. Biologically implausible data [3] were defined as >15,000 cpm. Non-wear-time was defined as a period of at least 60 consecutive minutes of zero counts, including up to 2 min of counts between 0 and 100 [20,21]. These records were removed from the analysis. A valid day was defined as a day of accelerometry with 10 or more hours of wear-time [22,23]. Daily wear-time was estimated by subtracting non-wear-time from 24 h of that day. The final sample consisted of children with at least four valid days including at least one valid weekend day. A consensus in the literature is yet to be reached regarding validated cut points for children’s physical activity [24]. Accelerometry data cut-off points used in the literature using the pediatric population are often derived from calibration studies providing counts per minute (cpm) equivalents to METs and have ranged from <100 to <1100 cpm depending on the device used. The purpose of this study was to estimate the associations that both the perceived and the researcher-rated BE have with children’s PA and sedentary time. It was not the objective of this study, however, to estimate the prevalence of physical activity or sedentary time, which might vary with the slightly difference cut points used. Therefore, activity level cut points were defined as follows: ST ≤ 150 counts per minute (cpm), not including sleep, light (LPA, 150–1951 cpm), moderate (MPA, 1952–5723 cpm), and vigorous PA (VPA, ≥5724 cpm) determined by the evolving evidence base on cut points for pediatric populations [25]. Moderate-to-vigorous physical activity (MVPA) ≥1952 cpm) was calculated as the total minutes of MVPA divided by the number of days of valid wear.

### 3.2. Measurement of Built Environment

In implementing NALP tools, pairs of assessors independently rated neighborhoods’ BE by travelling a predetermined route created by random selection and connection of block segments to determine a walking route [17]. Similarly, two observers were employed to administer the IMI audit tool to measure neighborhood block segments based on BE in five dimensions: diversity of destinations, pedestrian access, attractiveness, safety from traffic, and safety from crime [17]. NALP consists of 22 items within four areas: Activity Friendliness, Safety, Density of Destinations, and Universal Accessibility. Using this method, assessors rated each item on a 6-point scale after walking a pre-defined route in each neighborhood that connected 10 randomly selected street segments. IMI consists of a 229-item inventory of neighborhood features within five areas (on a 10-point scale): Attractiveness, Diversity of Destinations, Pedestrian Access, Safety from Traffic, and Safety from Crime. Twenty percent of street segments in each neighborhood were randomly selected and observed. Each segment consists of the two facing sides of a street block. The NALP tool takes into account the impression of the entire neighborhood based on the systematic observations of the researchers. The IMI is based on the detailed observations of each individual segment, an audit. The items in the IMI tools were tested for inter-rater reliability in both southern California and the Minneapolis—St. Paul metropolitan area samples [26]. Inter-rater reliability was high, with 77 percent of the items attaining 80% agreement or better in both southern California and Minnesota reliability tests. The predictive validity of the inventory was also tested [27]. NALP was validated by the SCHK team by adding a new dimension called universal accessibility (which measures disabled individuals’ access to BE) to existing dimensions of safety, density of destinations, and activity friendliness [28]. The items in NALP tools have a good reliability and convergent validity and are able to capture between neighborhood differences [18]. Survey data were collected using an SASK questionnaire on children’s perceptions of their neighborhood BE and demographics at three different time frames over a 1-year period between September 2014 and June 2015. Items addressed various aspects of the child perceived BE attributes, such as activity friendliness, walkability, accessibility and availability of parks and recreation facilities, and safety. For example, children were asked whether they had parks in their neighborhood (dichotomous response, yes or no) and if they observed any criminal activity (dichotomous response, yes or no) in their neighborhood (see Supplementary File). Since we were interested in combining both perceptions and researcher-rated BE measures (which were measured at a single time point) and including them in the model, we used the average perceived BE measures in this analysis.

### 3.3. Covariates

Individual level variables were used to account for factors specific to each child that may influence their physical activity. These variables include (with the reference category italicized): sex (male versus female); age in years (continuous); and family annual household income. Household income was collapsed from a 7-level to a 4-level categorical variable: $20,000 or less (reference); $20,001–$60,000; $60,001–$100,000; more than $100,000; and chose not to answer/unknown. The focus of this study was to examine the associations between the perceived and researcher-rated measured neighborhood BE characteristics and PA and ST, controlling for the effects of well-known covariates [29] such as child’s age, sex, and annual household income (parent reported). We were interested in whether associations between perceived and researcher-rated BE measures and PA and ST were modified by the child’s age, sex, and household income. Thus, selected effect modifications involving each of the three demographic factors were tested and none were significant at *p* < 0.5, therefore, they are not reported.

### 3.4. Statistical Analysis

Statistical analyses were performed with SPSS, version 25, software [30]. Using linear mixed-effects models, a multilevel modeling approach was taken to estimate the association between attributes of the neighborhood BE and the average daily minutes of PA and ST outcomes (clustered by child and neighborhood) [31]. A series of models using stepwise linear regression analyses with backward elimination were specified to assess the associations between the dependent variables (min/day MVPA, LPA, and ST) and the explanatory variables (e.g., the children’s perceived and researcher-rated BE attributes). All models included a random intercept to account for the baseline responses and a random time slope to account for within- and between-participant variability. Each BE attribute was entered one at a time with the dependent variable, and any BE variable removed sequentially that met the criterion for elimination (*p* ≥ 0.20), culminating in a final model incorporating multiple BE covariates. This procedure was repeated until the neighborhood BE attributes with a *p* < 0.20 remained in bivariable analysis to become candidates for the multivariable model. Significant variables were subsequently assessed for multicollinearity using Variance Inflation Factors (VIFs) prior to development of a final model. Assessment of VIFs indicated that collinearity was not problematic (all VIFs < 2), so all candidate variables from bivariable analysis were entered the final model. We investigated each outcome in separate models and retained variables with *p*-values of less than 0.05 in the final model. A final model was developed with the neighborhood BE characteristics that demonstrated a significant association with physical activity outcomes (MVPA, LPA, and ST). Selected effect modifications involving each of the three demographic factors (child’s age, sex, and family household income) were tested and none were significant at *p* < 0.05. Coefficients from the final model were used to compare the statistically significant perceived and researcher-rated measures of BE with MVPA, LPA, and ST.

## 4. Results

Table 1 reports the characteristics of the participating children. The participants were on average 10.97 ± 0.01 years old and majority of them were between 10 and 11 years old (71.2%). Over the one-year collection period, participants accumulated a daily mean of 39.4 MVPA, 335.4 LPA, and 271.4 ST minutes/day. Males accumulated significantly more MVPA, but significantly less LPA, in comparison to females. Older children accumulated significantly less MVPA and LPA, but significantly more ST. ST did not differ by sex, however, when a child’s entire day was considered (Table 1).

Within the study population, the category “Unknown” for annual household income includes those who actively chose not to answer, did not know their annual household income, or did not provide an answer.

In the fully adjusted models (Table 2), we observed a positive, statistically significant association between perceived availability and accessibility of parks and other recreational facilities and MVPA (B = 2.7; 95% confidence interval, CI: 1.4, 4.1). Children’s perception of the absence of sidewalks and their reporting concern for crime were associated with 2.6 and 1.8 fewer minutes of MVPA, respectively. We observed some mixed evidence for the association between researcher-rated BE and MVPA, and furthermore, some of these associations were in unexpected directions. As expected, researcher-rated omnibus neighborhood safety (from the NALP) was associated with higher levels of MVPA (B = 3.9, 95% confidence interval, CI: 2.9, 4.9). However, opposite to what we expected, researcher-rated specific measure of safety from crime (from IMI) was associated with lower levels of MVPA (B = −1.1, 95% confidence interval, CI: −1.5, −0.7) (Table 2).

Children who perceived that their neighborhoods were not places with social and physical disorder (e.g., an absence of threats such as dogs or strangers in the neighborhood) were engaged in 6.1 more min of LPA year-round. Children who reported the presence of role models who were active in their neighborhood (e.g., seeing others do exercise) were engaged in 22.2 more min of LPA. Contrary to our hypothesis, researcher-rated neighborhood safety (NALP) and safety from crime (IMI) showed negative, significant associations with LPA (each unit increase in scores on NALP safety and IMI safety from crime was associated with 10.0 and 4.4 fewer min of LPA, respectively) (Table 3).

Children’s reports of a higher perceived availability of parks and other recreational facilities in their neighborhood was associated with lower levels of ST year-round. Children who indicated a perceived absence of sidewalks that limit their opportunities to walk/bike in the neighborhood had 10.4 more min of ST. Children who reported that they have adult role models who were active in the neighborhood, had 25.0 fewer min of ST. As expected, researcher-rated activity friendliness (NALP) was associated with decreased ST, and so was pedestrian accessibility (IMI). However, once again unexpectedly, the specific measure of safety from crime (IMI) was associated with an increased in ST (B = 8.8, 95% confidence interval, CI: 6.2, 11.5) (Table 4)

Some consistent patterns are found within all models estimating the association between different intensity levels of PA, ST, and neighborhood BE attributes (Table 5). For example, children’s reports of the availability of parks and other recreation facilities was associated with higher levels of MVPA and lower levels of ST. Similarly, children’s reports on having active role models in their neighborhood (seeing other adults and adolescents being physically active) was associated with higher levels of LPA and lower levels of ST. In addition, children’s reports on low pedestrian infrastructure and accessibility (e.g., no sidewalk) was associated with lower levels of MVPA and higher levels of ST. Surprisingly, but consistently, researcher-rated safety from crime (IMI) was associated with lower levels of PA (both MVPA and LPA) and higher levels of ST. It is interesting to note, however, that children in neighborhoods measured as safe from crime using an IMI audit tool had lower MVPA, while children in neighborhoods measured as generally safe using an NALP tool had a higher MVPA.

## 5. Discussion

This paper provides an understanding of the perceived or researcher-rated (by independent raters) neighborhood BE characteristics associated with childhood physical activity and sedentary time over an entire school year. This study contributes to existing knowledge on how the perceptions of children and the actual built environment affect their PA behaviors. Although researcher-rated measures provide the necessary rigor to BE measurement, there is evidence to suggest that environmental perceptions may be equally predictive of physical activity. To date, as relatively little research linking built environments and physical activity behavior has been done by taking both researcher-rated and perceived measures of BE into account, this study thus provides the evidence to include both researcher-rated and perceptual measures of BE in future activity behavior research in children.

This study shows that perception (perceived BE factors) are more salient than researcher-rated measures of BE in shaping children’s PA and ST. Most perceived BE features consistently predicted PA and ST across the entire year. That is, children’s reports (perceived) of the presence of parks and other recreational facilities, pedestrian amenities, and active role models in the neighborhood were associated with higher levels of PA and lower levels of ST. Children’s perceived concerns for crime were associated with lower levels of MVPA however, and had no impact on ST.

These findings add to and extend the existing evidence that positive perceptions of neighborhood BE features increase time spent in higher intensity PA among school-aged children [11,12,13,32]. Our finding that children’s reports of the presence of parks predicted a higher accumulation of time in PA in children corroborates with existing literature [11,12,33], although not all studies find this positive association [34]. In their review article, Davison and colleagues demonstrated a positive association between children’s reports of accessibility and availability of parks and recreational facilities with physical activity levels in children [12]. A recent cross-sectional study among four hundred children aged 9–14 years demonstrated that children from neighborhoods with greater access to parks with sports fields and multi-use paths accumulated significantly higher levels of accelerometer-measured MVPA [35]. Conversely, Timperio et al. found that children who perceived that their neighborhood had no suitable parks or sports grounds near home had a lower likelihood of walking or cycling in the neighborhood [36]. The perception of a lack of pedestrian facilities (e.g., an absence of sidewalks) predicted a higher accumulation of ST in children, which is also consistent with the current literature. Jago et al., [13], in one of the few studies to examine both PA and ST, found that good sidewalks were negatively associated with minutes of sedentary behavior and positively associated with minutes of light-intensity physical activity. However, Mota et al. [37] found no association between perceived availability and quality of sidewalks and cycling infrastructure and adolescents’ self-reported activity.

Our finding that children’s reported safety concerns about crime were associated with a lower accumulation of PA are in line with the existing evidence that perceived safety is an important determinant of PA levels among school-aged children [32,38]. One Canadian study based in Saskatoon found that neighborhood streets or paths where children can walk and cycle without feeling threatened, parks and green spaces free of criminal activity, and neighborhoods where people know each other facilitated children’s physical activity [39]. Molnar et al., [40] evaluated children’s perceptions of the neighborhood as a safe place to play and found that the perception of the neighborhood as being “unsafe to play” in was negatively associated with time spent engaged in recreational physical activity. Burdette et al., [10] reported that young children who live in neighborhoods perceived to be the least safe were more likely to watch more than 2 h of TV per day. However, as important as the perceptions of safety for children’s activity levels or time spent in sedentary appear to be, more research is needed in order to understand how perceptions of safety affect children’s activity behavior. Future research should consider examining specific aspects of safety (e.g., crime, bullying, traffic), its context, including influences from parents and friends, and their impact on physical activity and sedentary behavior.

Overall, fewer researcher-rated BE characteristics predicted PA outcomes, and among those that did, no single feature of the BE, except for the measured safety from crime (IMI), consistently predicted PA outcomes. Even though NALP safety was positively associated with MVPA, IMI safety from crime was surprisingly, but consistently, associated with decreased MVPA and increased ST. One possible explanation for this unexpected finding is the nature (item constitution) of the NALP, an omnibus tool, versus the IMI, which is an audit tool. The NALP tool considers the impression of the entire neighborhood based on the systematic observations of the researchers, whereas the IMI is based on direct observations of each individual segment in a neighborhood. Thus, the measure of general safety from NALP, relates better to the findings of children’s perception of safety (from crime) and the levels of MVPA. In line with these findings, Uys et al., [41] reported an inverse association between researcher-rated safety (from crime) and adolescent’s after-school MVPA. Our finding that researcher-rated pedestrian accessibility and activity friendliness predicted lower accumulation of ST in children is consistent with the literature [10,42,43]. For example, Wilson et al., [43] reported that greater accessibility was associated with fewer minutes of ST in children. These findings suggest that both researcher-rated and perceived BE may have different effects and/or independent roles in shaping youth PA and ST, while perceptions may play a much larger role than the researcher-rated environment in predictions of children’s PA behavior.

This study is unique as it includes device-based measures of children’s physical activity behavior, survey data exploring children’s perception of the BE characteristics, and researcher-rated neighborhood level BE characteristic data. While subjective measures present limitations in capturing physical activity due to poor reliability and validity, participant recall bias, and interpretation of questions, this study is strengthened by its employment of accelerometer-based measures in capturing physical activity behaviors, thus avoiding self-report bias. Even though objective measures (e.g., accelerometer) have some component of measurement error [44], there is no evidence supporting the effect of any systematic bias (e.g., under estimation or over estimation) on introducing bias in the association between the environment and the behavior. The approach taken in this study to combine perceptual and researcher-rated measures of BE and their relative effects on children’s activity behavior is a key strength of the study. Other strengths include the large representative sample of children aged 9 to 14 years representing all socioeconomic categories within all types of neighborhoods built using a range of urban design forms (grid-pattern, mixed-grid, curvilinear road networks), the use of data over a year that better captures children’s PA in a typical week, and the use of multilevel mixed-effect models to account for the clustering of children within schools and schools within neighborhoods across Saskatoon. It is important to note that even though the SASK study had longitudinal data, this current study used these data cumulatively and not longitudinally. There are alternative ways (e.g., buffers around children, activity space) to define child-centered neighborhoods. Nevertheless, this study focused on neighborhoods defined by the city (i.e., municipal boundaries, development era, and associated urban design) that in both types of measures refer to the same neighborhood. Though both perceptual and researcher-rated BE measures are about the same neighborhood, these measures were collected in two different time periods 2–4 years apart. We acknowledge that both types of BE measures collected in the same year would have been ideal, but we expect that neighborhood characteristics used in this study will have not changed meaningfully over a period as short as 2 to 4 years.

Although only a few studies have quantified the agreement between perceived and researcher-rated environmental measures (mostly in adult population), future studies should investigate whether the agreement between the measures differ across neighborhood and individual characteristics to help further define the relationship between the two and potentially lead to PA promotion strategies that target such groups. Differences in the way children and parents perceive the safety and other environmental features in their physical and social neighborhood environment can in turn impact their health behaviors (e.g., physical activity). Further research should also identify, however, any differences that may exist between levels of children’s PA, from the mismatch between young children’s and parents’ perceptions of the neighborhood BE. The generalizability of specific results is limited only in so far as other communities and children’s similarity to Saskatoon.

## 6. Conclusions

This study examines how perceived and researcher-rated BE characteristics are associated with children’s PA and ST outcomes. Many results are consistent with the findings of previous studies, providing further support for policies that promote child-friendly neighborhoods to support physical activity. This study shows that perceived BE factors are more strongly associated with children’s PA and ST than that of researcher-rated measures of BE. Therefore, it may be important that future research efforts looking at BE and PA/ST include perceived factors of BE, and do not just rely on researcher-rated measures of BE.

## Figures and Tables

**Table 1:** Study population characteristics of those contributing valid accelerometer data (*n* = 619).

Study Population Characteristics		*n* (%)	Mean Daily Moderate to Vigorous Physical Activity (MVPA) (min/day)	ANOVA *p*-Value	Mean Daily Light Physical Activity (LPA) (min/day)	ANOVA *p*-Value	Mean Daily Sedentary Time (ST) (min/day)	ANOVA *p*-Value
**Sex**	Male	256 (41.3)	42	<0.001	324	<0.001	274	0.97
	Female	363 (58.6)	38		350		273	
**Age (years)**	9	29 (4.7)	43	<0.001	369	<0.001	242	<0.001
	10	214 (34.6)	42		351		247	
	11	195 (32.2)	39		339		275	
	12	105 (17.1)	38		320		301	
	13–14	76 (11.4)	35		313		316	
**Annual Household Income**	Less than $20,000	12 (1.9)	39	0.85	311	0.05	293	0.35
	$20,000 to $60,000	86 (13.9)	38		341		270	
	$60,000 to $100,000	104 (16.9)	39		346		261	
	$100,000 or more	258 (41.8)	40		332		275	
	Unknown	157 (25.4)	41		333		271	

**Table 2:** Associations between BE characteristics and average daily minutes of MVPA (only significant associations from the final model presented).

Built Environment Characteristics	MVPA (min/day)			
	Unadjusted Models		Adjusted Models	
	B (SE)	95% CI	B (SE)	95% CI
**Perceived Neighborhood-built Environment Attributes**				
Absence of a sidewalk	−2.8 (0.7) **	−4.2, −1.5	−2.6 (0.7) **	−3.9, −1.3
Low traffic volume	-	-	-	-
Absence of social/physical disorder (no unattended dogs/unsafe people)	-	-	-	-
Availability of parks/facilities	2.2 (0.7) **	0.8, 3.6	2.7 (0.7) ***	1.4, 4.1
Presence of street lighting	-	-		
Concerns for crime	−1.6 (0.7) *	−2.9, −0.2	−1.8 (0.7) *	−3.2, −0.4
Positive social interaction/active role models	-	-	-	-
**Researcher-rated Neighborhood-built Environment Attributes**				
NALP activity friendliness	-	-	-	-
NALP density of destinations	-	-	-	-
NALP safety	5.2 (0.7) **	3.8, 6.5	3.9 (0.5) ***	2.9, 4.9
NALP universal accessibility	-	-	-	-
IMI attraction	−3.2 (0.6) *	−4.4, −1.9	−2.2 (0.5) *	−3.2, −1.2
IMI density of destinations	-	-	-	-
IMI safety from crime	−1.1 (0.3) ***	−1.7, −0.5	−1.1 (0.2) ***	−1.5, −0.7

Main effect model (unadjusted): Neighborhood built environment (BE) characteristics that demonstrated statistically significant associations with the average daily minutes of moderate-to-vigorous physical activity (MVPA) at *p* < 0.05. Fully adjusted model: Neighborhood characteristics that demonstrated statistically significant associations with the average daily minutes of MVPA at *p* < 0.05, controlling for demographic variables (child’s age, sex, and family annual household income). * *p* < 0.05; ** *p* < 0.01; *** *p* < 0.0001.

**Table 3:** Associations between BE characteristics and average daily minutes of LPA (only significant associations from the final model presented).

Built Environment Characteristics	LPA (min/day)			
	Unadjusted Models		Adjusted Models	
	B (SE)	95% CI	B (SE)	95% CI
**Perceived Neighborhood-built Environment Attributes**				
Absence of social/physical disorder	8.1 (3.1) *	2.0, 14.0	6.1 (2.8) *	0.7, 11.5
Positive social interaction/active role models	20.5 (6.0) **	8.7, 32.4	22.2 (5.7) **	11.1, 33.3
**Researcher-rated Neighborhood-built Environment ttributes**				
NALP safety	−11.1 (3.2) ***	−17.4, −4.8	−10.0 (2.5) ***	−14.9, −5.1
NALP universal accessibility	-	-	-	-
IMI attraction	-	-	-	-
IMI safety from crime	−2.7 (1.3) **	−5.2, −0.1	−4.4 (1.0) **	−6.5, −2.3
IMI safety from traffic	-	-	-	-
IMI pedestrian access	-	-	-	-

Main effect model (unadjusted): Neighborhood characteristics that demonstrated statistically significant associations with the average daily minutes of LPA at *p* < 0.05. Fully adjusted model: Neighborhood characteristics that demonstrated statistically significant associations with the average daily minutes of LPA at *p* < 0.05, controlling for demographic variables (child’s age, sex, and family annual household income). * *p* < 0.05; ** *p* < 0.01; *** *p* < 0.0001.

**Table 4:** Associations between BE characteristics and average daily minutes of ST (only significant associations from the final model presented).

Built Environment Characteristics	ST (min/day)			
	Unadjusted Models		Adjusted Models	
	B(SE)	95% CI	B(SE)	95% CI
**Perceived Neighborhood-built Environment Attributes**				
Absence of a sidewalk	10.4 (4.2) *	2.1, 18.7	7.0 (3.9) *	−0.8, 14.8
Low traffic volume	-	-	-	-
Absence of social/physical disorder	-	-	-	-
Availability of parks/facilities	−16.9 (4.4) **	−25.5, −8.4	−16.4 (4.3) **	−24.8, −8.0
Presence of street lighting	-	-	-	-
Concerns for crime	-	-	-	-
Positive social interaction/active role models	−19.8 (7.9) **	−35.2, −4.1	−25.0 (7.1) **	−38.8, −11.0
**Researcher-rated Neighborhood-built Environment Attributes**				
NALP activity friendliness	−9.7 (2.6) *	−14.9, −4.5	−6.8 (2.4) *	−11.6, −2.0
NALP density of destinations	-	-	-	-
IMI attraction	-	-	-	-
IMI density of destinations	-	-	-	-
IMI safety from crime	9.3 (1.9) ***	5.5, 13.0	8.8 (1.3) ***	6.2, 11.5
IMI safety from traffic	-	-	-	-
IMI pedestrian access	−6.2 (3.2) **	−12.6, 0.1	−7.9 (2.8) *	−13.3, −2.5

Main effect model (unadjusted): Neighborhood characteristics that demonstrated statistically significant associations with the average daily minutes of ST at *p* < 0.05. Fully adjusted model: Neighborhood characteristics that demonstrated statistically significant associations with the average daily minutes of ST at *p* < 0.05, controlling for demographic variables (child’s age, sex, and family annual household income). * *p* < 0.05; ** *p* < 0.01; *** *p* < 0.0001.

**Table 5:** Results across all PA levels and ST in relation to neighborhood BE characteristics.

Predictors (Built Environment Characteristics)	Physical Activity Outcomes		
	MVPA	LPA	ST
**Children’s Perceived BE**			
Availability of parks	2.7 more min/day	-	16.4 fewer min/day
Absence of sidewalks	2.6 fewer min/day	-	7.0 more min/day
Presence of physically active role models (see others do exercise)	-	22.2 more min/day	25 fewer min/day
Concerns for crime	1.8 fewer min/day	-	-
Absence of social/physical disorder	-	6.1 more min/day	-
**Researcher-rated BE**			
IMI safety from crime	1.1 fewer min/day	4.4 fewer min/day	8.8 more min/day
NALP safety	4 more min/day	10.0 fewer min/day	-
IMI attraction	2.2 fewer min/day	-	-
NALP activity friendliness	-	-	6.8 fewer min/day
IMI pedestrian access	-	-	7.9 fewer min/day

“-“ denotes no significant impact on PA levels and ST.

## Supplementary Materials

The following are available online at https://www.mdpi.com/1660-4601/17/11/3837/s1, Table S1: Descriptive statistics of neighborhood perceived, and researcher-rated BE measures, Table S2: Perceived BE variables used in the study and their component survey.
