# Supplementary material for: Neighborhood Built Environment Measures and Association with Physical Activity and Sedentary Time in 9–14-Year-Old Children in Saskatoon, Canada
Source: Int J Environ Res Public Health. 2020 May 28;17(11):3837. doi: 10.3390/ijerph17113837 (PMC7312779; doi:10.3390/ijerph17113837)
Supplement: Supplementary file 1 [file ijerph-17-03837-s001.pdf]

Supplementary:

Table S1. Descriptive statistics of neighborhood perceived, and researcher-rated BE measures.

Neighborhood Researcher-Rated BE Measures	Mean (S.D)	Different Neighborhood BE Features (Perception)	Yes (%)
NALP (scale 1–6)		Positive perception	
NALP activity friendliness	3.79 ± .49	Sidewalk	70.7
NALP density of destinations	3.72 ± .78	Street lighting	94.7
NALP safety	3.90 ± .39	Less traffic	86.6
NALP universal accessibility	2.18 ± .64	Road safety	90.1
IMI (scale 1–10)		Parks	89.9
		PA role models	96.8
		Negative perception	
IMI attraction	4.82 ± .34	Scary dogs	33.9
IMI diversity of destinations	5.34 ± 2.56	Road crossing difficulties	93.1
IMI pedestrian accessibility	5.17 ± .46	Criminal activities	92.2
IMI safety from crime	8.72 ± .94	Hilly roads	88.5
IMI safety from traffic	6.32 ± .88		

Description of BE audit tools: NALP consists of 22 items within four areas: Activity Friendliness, Safety, Density of Destinations, and Universal Accessibility. Using this method, observers rated each item on a 6-point scale after walking a pre-defined route in each neighborhood that connected 10 randomly selected street segments. IMI consists of a 229-item inventory of neighborhood features within five areas (on a 10-point scale): Attractiveness, Diversity of Destinations, Pedestrian Access, Safety from Traffic, and Safety from Crime. Twenty percent of street segments in each neighborhood were randomly selected and observed. Each segment consists of two facing sides of a street block and is indicated by a numbered flag on the map. The NALP tool is more subjective in nature and it takes into account the impression of the entire neighborhood based on the systematic observations of the researchers. In contrast, the IMI is more objective in nature and is based on observations of each individual segment.

Descriptive statistics:

Researcher-rated BE Measures: For example, out of a possible highest rating of 10, neighborhoods in Saskatoon, Canada, on average, rated as 8.72 for safety from crime (IMI). Out of a possible highest rating of 6, neighborhoods in Saskatoon, on average rated 3.90 for Safety (NALP).

Perceived BE Measures: For example, 70.7% of children report that their neighborhoods have sidewalks (in terms of positive perception). In contrast, 33.9 % children report that they observed scary dogs in their neighborhoods which they find a safety issue (in terms of negative perception).

Table S2. Perceived BE variables used in the study and their component survey.

Survey Component	Variables Recoded/Used in the Study
Sidewalk (yes/no)	Same
Less traffic (yes/no)	Same
Scary dog or people	Absence of social/physical disorder (no unattended/scary dogs/unsafe people)
Parks	Same
Street lighting	Same
Criminal activity	Concerns about crime
Crossing road safely	Road safety
Seeing others being physically active	Physical activity role model
